# Long-Term Response to Gemcitabine, Cisplatin, and Nab-Paclitaxel Followed by Maintenance Therapy for Advanced Gallbladder Cancer: A Case Report and Literature Review

**DOI:** 10.3389/fonc.2021.733955

**Published:** 2021-10-05

**Authors:** Ting Liu, Qing Li, Wenjie Zhang, Qing Zhu

**Affiliations:** ^1^ Department of Biotherapy, Cancer Center, West China Hospital, Sichuan University, Chengdu, China; ^2^ Cancer Center, West China Hospital, Sichuan University, Chengdu, China; ^3^ Department of Nuclear Medicine, West China Hospital, Sichuan University, Chengdu, China; ^4^ Department of Abdominal Oncology, Cancer Center, West China Hospital, Sichuan University, Chengdu, China

**Keywords:** gallbladder cancer, nab-paclitaxel, complete response, maintenance therapy, case report

## Abstract

**Background:**

Gallbladder cancer (GBC) is the most common and devastating tumor type of biliary tract cancer (BTC) with poor outcomes. A new combined regimen of gemcitabine, cisplatin, plus nab-paclitaxel is currently considered an effective option for patients with advanced BTC following the results of a phase II trial. In addition, maintenance therapy after first-line treatment has been shown to improve disease control rate of various solid tumors but has not been evaluated for GBC patients. The scenario we report herein is of a metastatic GBC patient treated with the triple-drug regimen followed by maintenance therapy with capecitabine or S-1, who achieved a long-term survival benefit.

**Case Presentation:**

A 68-year-old man was diagnosed with gallbladder adenocarcinoma with liver, supra-diaphragmatic, and abdominal lymph node metastases (cT3N2M1, stage IVB). Partial response (PR) was achieved after five cycles of gemcitabine and cisplatin chemotherapy. A further three cycles of nab-paclitaxel plus gemcitabine-cisplatin regimen yielded a complete response of all tumor lesions. Subsequent administration of maintenance therapy with capecitabine followed by S-1 achieved a disease-free survival of 15 months for the patient. Moreover, the patient remained responsive to this triple-drug regimen when the disease progressed, achieving PR after two cycles of chemotherapy. Overall, the treatment regimens were well tolerated with no grade 3 or higher adverse effects occurring. Notably, the serum carbohydrate antigen 199 (CA199) levels were closely related to the treatment response and increased before the lesions were found on PET-CT during follow-up.

**Conclusion:**

Our findings suggested that adding nab-paclitaxel into gemcitabine-cisplatin regimen may result in a favorable efficacy in patients with advanced GBC. Further maintenance therapy with capecitabine or S-1 after first-line therapy appeared to be a reasonable option for these patients, and it is valuable to monitor CA199 levels during treatment and follow-up.

## Introduction

Biliary tract cancer (BTC) is a rare but invasive malignancy including intrahepatic cholangiocarcinoma (ICC), extrahepatic cholangiocarcinoma (ECC), and gallbladder cancer (GBC), of which GBC is the most common and lethal tumor type, accounting for approximately 60%–70% of cases (1–3). Routine cholecystectomy has reduced the incidence of GBC, with 219,420 new cases and 165,087 associated deaths being estimated worldwide in 2018 (4–6). Radical surgery is the cornerstone for curing GBC patients. However, most GBC patients present at an advanced stage and miss the opportunity for surgical treatment and even undergoing radical surgical treatment, and more than 60% of patients still experience tumor recurrence (7, 8). The gemcitabine-cisplatin regimen is currently the standard first-line therapy engaged in advanced BTC patients with a median overall survival (OS) of less than 1 year (9). Consequently, it remains imperative to explore new treatment modalities to improve the prognosis of these patients.

Preclinical and clinical trials have illustrated that nab-paclitaxel increased the intra-tumor concentration of gemcitabine by decreasing the gemcitabine metabolizing enzyme, cytidine deaminase (10, 11). Based on the synergistic antitumor effects of nab-paclitaxel and gemcitabine, the combined regimen was proved suitable as first-line treatment option for advanced pancreatic cancer (12). A phase II, single-arm trial has for the first time demonstrated that adding nab-paclitaxel to gemcitabine-cisplatin therapy prolonged progression-free survival (PFS) and OS for patients with advanced BTC (13); more evidence is needed to confirm this conclusion. In addition, maintenance therapy has been demonstrated with favorable survival benefits in a variety of solid tumors (14–18). Nevertheless, it is unclear whether patients with advanced GBC should receive maintenance therapy after first-line treatment. Furthermore, no reliable tumor markers are available to monitor the status of GBC patients who are vulnerable to recurrence and metastasis. Herein, we presented a case of an advanced GBC patient who achieved a complete response (CR) and long-term disease-free survival (DFS) after treatment with nab-paclitaxel plus gemcitabine-cisplatin followed by maintenance therapy with capecitabine or S-1. Furthermore, we reported that serum carbohydrate antigen 199 (CA199) levels were closely related to the treatment response throughout the treatment process.

## Case Description

**Figure 1:**
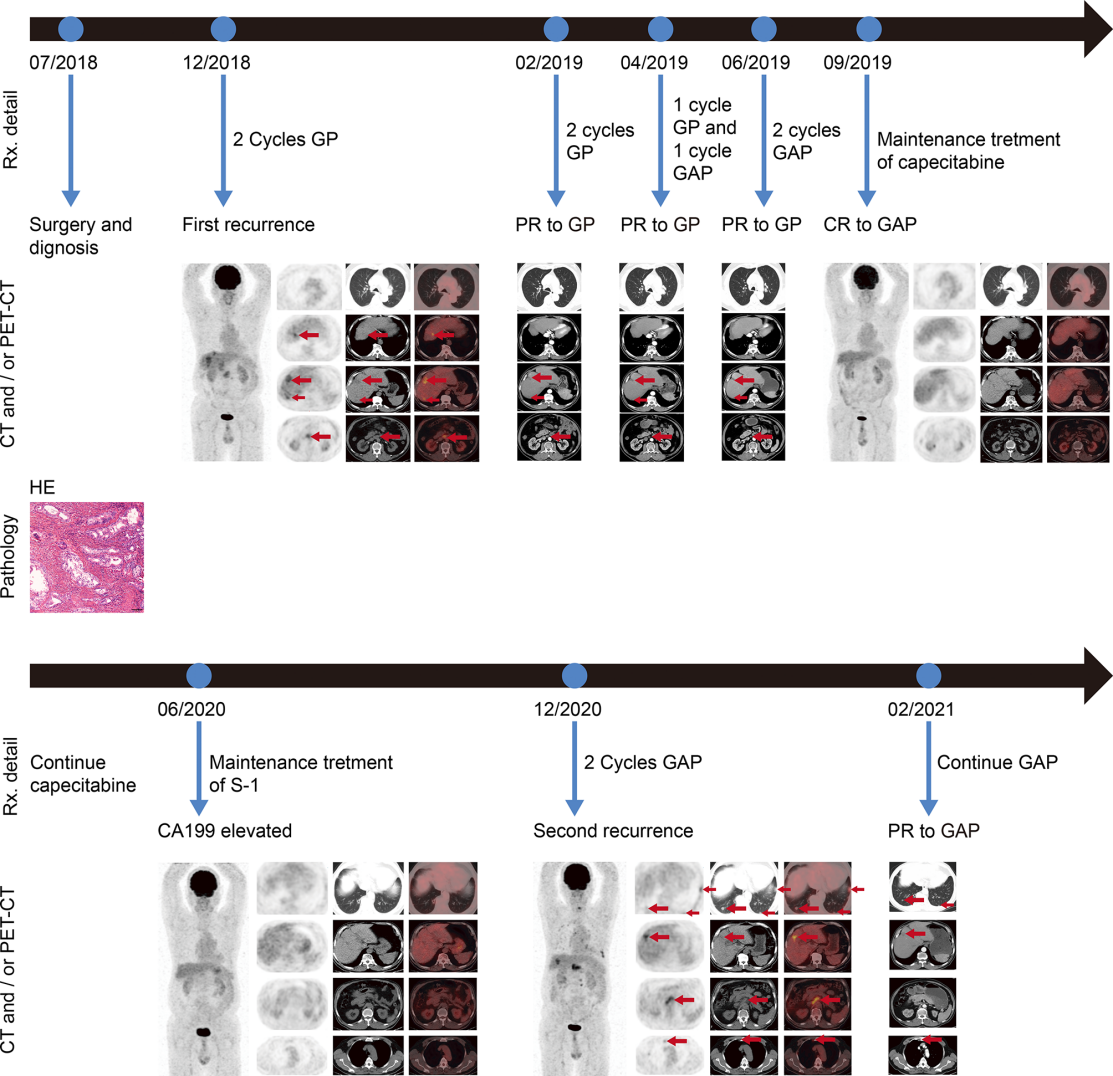
Imaging scans, pathological findings, and timeline with relevant data from the episode of care. HE staining of the postoperative pathological specimen indicated adenocarcinoma (scale bars represent 100 µm). Before first-line treatment, representative PET-CT images were shown. Regular revision computed tomography (CT) was used to assess treatment efficacy during first-line treatment. After five cycles of gemcitabine and cisplatin chemotherapy followed by three cycles of nab-paclitaxel in combination with gemcitabine and cisplatin chemotherapy, representative PET-CT images were shown. No lesions were detected by PET-CT, when an elevated CA199 was tested. When the patient began to experience progressively worsening abdominal pain and an abnormal increase in CA199, PET-CT was performed and representative images were shown. CT scan was performed after two cycles of nab-paclitaxel plus gemcitabine and cisplatin chemotherapy, and showed a PR of all tumor lesions. Rx, treatment; PET-CT, positron emission tomography–computed tomography; CT computed tomography; CA199, cancer antigen 199; PR partial response; CR, complete response; G, gemcitabine; A, nab-paclitaxel; P, cisplatin.

**Figure 2:**
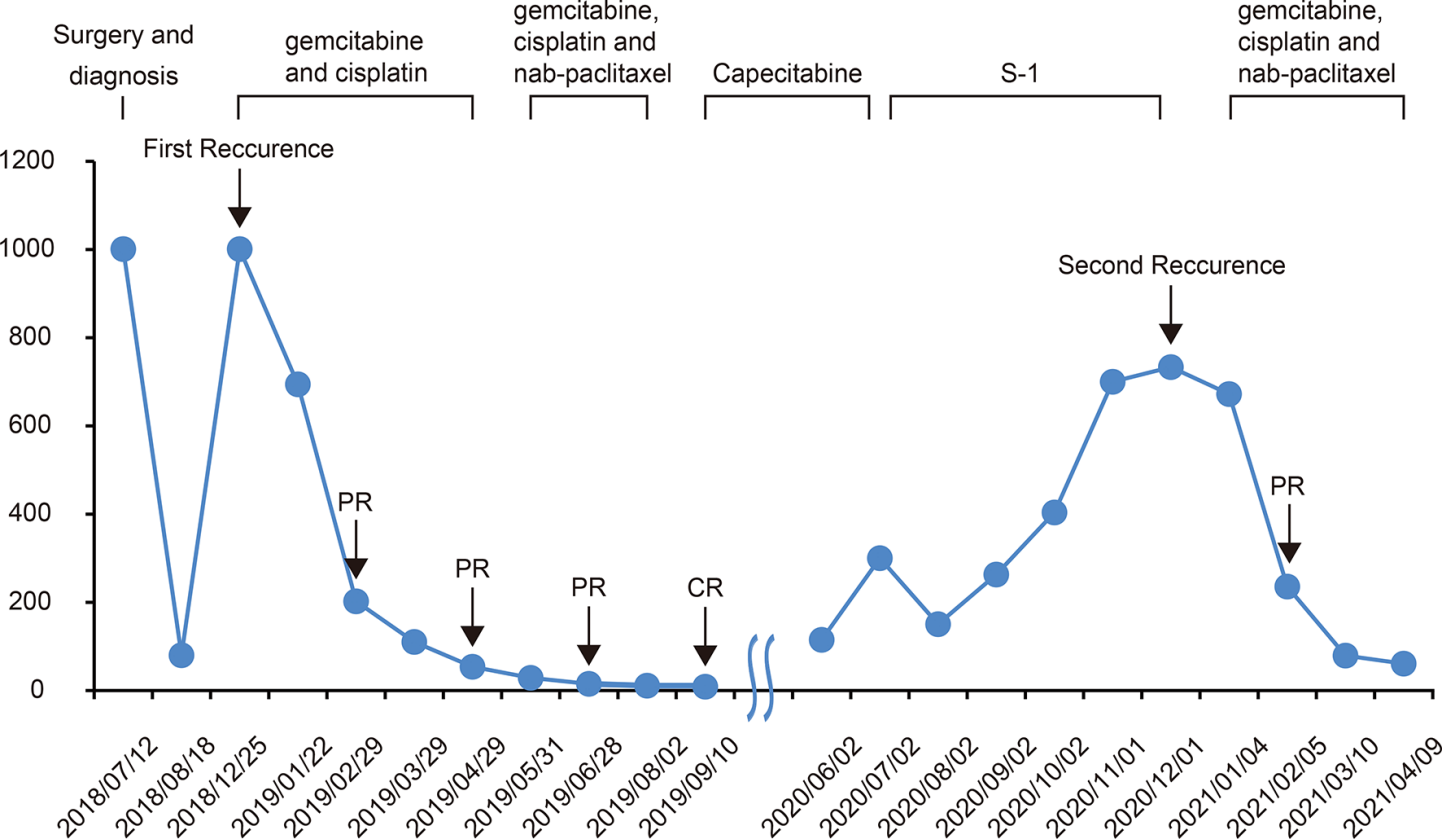
Changes in CA199 levels during the treatment and follow-up. The CA199 tumor marker was measured in patients’ blood at periodic intervals throughout the clinical course and annotated with date, therapeutic approach, and treatment efficacy. CA199, cancer antigen 199; CR complete response; PR partial response.

In November 2018, a 68-year-old man was admitted to our center with chief complaints of a significant increase in CA199 (>1,000.00 U/ml) without any other discomfort. Four months prior, he had been clinically diagnosed with GBC due to an elevated CA199 (>1000.00 U/ml) and gallbladder occupancy found in his physical examination. He underwent radical surgery at a local hospital. His postoperative pathology confirmed the diagnosis of GBC, and suggested a stage of pT3N1M0, stage IIIB according to the National Comprehensive Cancer Network staging criteria (**Figure 1**). After surgery, the serum CA199 level decreased to 80 U/ml (**Figure 2**). The patient did not undergo any postoperative adjuvant therapy. He had been generally fit, except for a 10-year history of hypertension and used propranolol for high blood pressure, which was well controlled. His Eastern Cooperative Oncology Group (ECOG) performance status on admission was 1. In December 2018, a fluorodeoxyglucose (FDG)-positron emission tomography computed tomography (PET-CT) at our hospital revealed multiple masses in the liver, extensive lymph nodes enlargement involving the anterior abdominal aorta, lesser omentum, and anterior supra-diaphragmatic, all with high FDG accumulation; and multiple nodules in the lung were considered inflammatory nodules (**Figure 1**). Detection of tumor markers showed that CA199 was >1,000.00 U/ml and carcinoembryonic antigen (CEA) was 21.63 ng/ml (**Figure 2**). A pathological consultation was performed in our hospital, and pathological diagnosis of adenocarcinoma was established. He was diagnosed with gallbladder adenocarcinoma with liver, supra-diaphragmatic, and abdominal lymph node metastases (cT3N2M1, stage IVB).

In December 2018, the patient received gemcitabine-cisplatin chemotherapy and experienced tolerable toxicities of reversible grade 2 anemia and grade 2 pruritic rash on the trunk and limbs. After two cycles of chemotherapy, his tumor shrank remarkably, and an objective response of partial response (PR) was assessed. Therefore, he continued to receive chemotherapy, and the tumor response was maintained after three additional cycles of chemotherapy. The tumor markers CA199 and CEA dropped to 53.94 U/ml and 1.96 ng/ml, respectively. In June 2019, based on the results of a phase 2 clinical trial, his chemotherapy regimen was switched to nab-paclitaxel plus gemcitabine-cisplatin regimen and experienced recurrent mouth ulcers in addition to hair loss plus slight leukopenia. The oral ulcers ameliorated with salt water gargling and the leukopenia was alleviated by an injection of colony-stimulating factor. After three cycles of the triple-drug chemotherapy, a CT scan showed disappearance of the tumor lesions, and PET-CT demonstrated no lesions in the liver and upper abdominal lymph nodes; the anterior group of lymph nodes in the diaphragm was smaller than before, and no increase in glucose metabolism was observed (**Figure 1**). Moreover, CA199 dropped to the normal range of 8.65 U/ml and CEA was 2.74 ng/ml (**Figure 2**). The patient achieved a CR to therapy. Then, he received 10 months of capecitabine monotherapy maintenance treatment.

**Figure 3:**
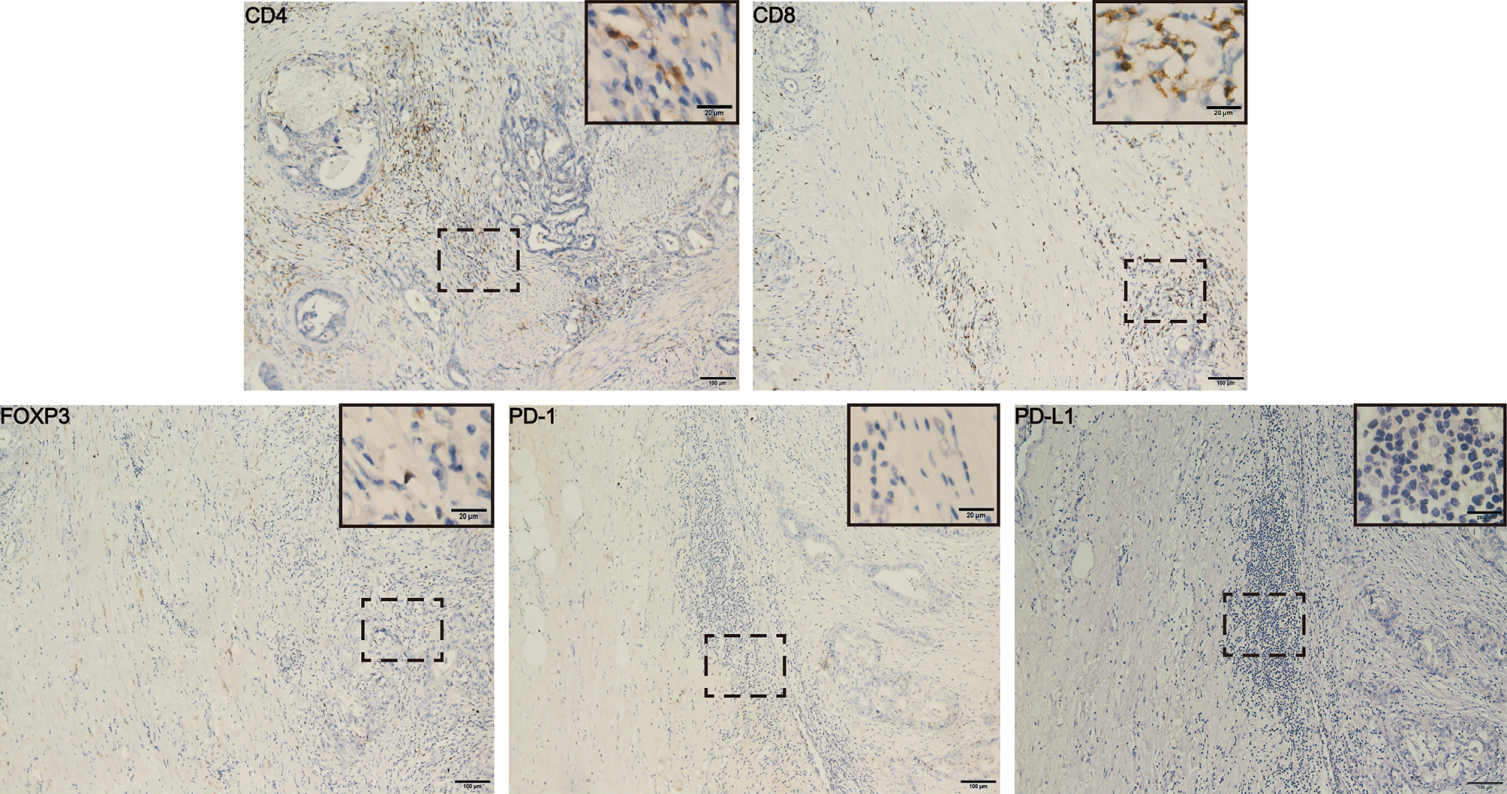
Immunohistochemical results from post-operative pathological tissues of the patient. The results showed higher number of CD4^+^ and CD8^+^ T cells and the lower number of FOXP3^+^ Tregs and PD-(L)1 expression in tumor microenvironment. Scale bars represent 100 µm (20 µm in the inset images). FOXP3, Forkhead box P3; PD-L1, programmed cell death-ligand 1.

In June 2020, his CA199 was abnormally elevated to 115 U/ml, while no other hematological abnormalities were observed, and no lesions were detected by PET-CT (**Figure 1**). Due to the adverse events of nausea and vomiting while on capecitabine, his maintenance regimen was converted to S-1 in July 2020, and he began to experience progressively worse epigastric pain and signs of epigastric tenderness after 4 months, with an increase in CA199 and CEA to 733.40 U/ml and 7.52 ng/ml, respectively. Subsequently, PET-CT, conducted in December 2020, showed multi-metastatic lesions in both lungs and liver, along with tumor metastases in the right parasternal, parietal trunk, and para-aortic lymph nodes (**Figure 1**). The patient received triple-drug regimen of nab-paclitaxel plus gemcitabine-cisplatin again. After two cycles of chemotherapy, an enhanced CT revealed significant downsizing of the tumor lesion with an evaluation of a PR efficacy (**Figure 1**). The CA199 and CEA levels also gradually decreased (**Figure 2**). Currently, the patient is still receiving regular triple-drug chemotherapy and his disease is well controlled. We further conducted immunohistochemical (IHC) staining of post-operative pathological specimens revealing the presence of a limited amount of programmed death 1 (PD-1) and programmed death ligand 1 (PD-L1) expression in the tumor microenvironment. In addition, compared to Forkhead box P3 (FoxP3)^+^ regulatory T cells (Tregs), the number of CD8^+^ cytotoxic T lymphocytes and CD4^+^ T helper lymphocytes was higher in the tumor microenvironment (**Figure 3**).

## Discussion

Chemotherapy regimens based on gemcitabine or fluorouracil are currently regarded as the recommended first-line treatment for patients with advanced BTC, but with limited efficacy (9, 19–21). It remains a necessity to explore new treatment options to improve the survival benefit for advanced BTC patients. Nab-paclitaxel is an albumin-bound paclitaxel granule formulation in which the albumin component binds to secreted protein, acidic and cysteine-rich (SPARC) (22). SPARC is secreted by peritumoral fibroblasts and is overexpressed in many advanced cancers, including biliary tract tumors, and binding to albumin sequesters albumin-bound paclitaxel to increase the intratumoral drug concentration (23). In addition, previous studies have confirmed that nab-paclitaxel improved the intratumoral concentration of gemcitabine by reducing gemcitabine-degrading enzymes (10, 24), and the synergistic effect of nab-paclitaxel and gemcitabine has demonstrated promising antitumor efficacy and a manageable safety profile (11). In a single-arm phase II study with a small sample, Shroff et al. first administered nab-paclitaxel plus gemcitabine-cisplatin regimen to previously untreated advanced BTC patients, and achieved a prolonged median PFS and median OS of 11.8 months and 19.2 months, respectively (13). Based on the favorable outcomes of this study, in our case, after receiving five cycles of gemcitabine-cisplatin chemotherapy and achieving PR, the patient was treated with three cycles of triple-drug chemotherapy, whereby the patient surprisingly achieved CR. More importantly, the regimen remained effective when the disease progressed again, and the patient achieved a PR after two cycles of chemotherapy. Our findings support the good efficacy of the triple-drug combination regimen in advanced GBC patients. We await the results of a phase III randomized controlled trial (RCT) of gemcitabine and cisplatin in combination with or without nab-paclitaxel for patients with advanced BTC (ClinicalTrials.gov identifier NCT03768414).

Shroff et al. (13) demonstrated that gemcitabine-cisplatin plus nab-paclitaxel for the first-line treatment of advanced BTC patients was associated with higher adverse event (AE) rates versus historical gemcitabine-cisplatin regimen. Their study revealed that 58% of patients experienced grade 3 or higher AEs, which were more common in the high-dose group. However, *post hoc* analyses illustrated that the treatment efficacy was not significantly related to the starting dose, while tolerability was improved with reduced-dose treatment compared to the high-dose treatment. The main AEs during triple chemotherapy reported in this case were alopecia, oral ulcers, and additional leukopenia, with no grade 3 or higher AEs, which may be associated with the low doses of gemcitabine (800 mg/m^2^) and nab-paclitaxel (100 mg/m^2^) that we used. We speculated that comparable efficacy and lower AE rates may be derived from lower doses of gemcitabine-cisplatin plus nab-paclitaxel triple-drug therapy.

No consensus has been reached on the availability and protocol of maintenance therapy for patients with advanced GBC after the first-line treatment has achieved tumor regression. Theoretically, maintenance therapy should control disease progression and prolong survival benefit; however, extending first-line chemotherapy until disease progression is unrealistic due to the accumulating toxic effects of chemotherapy drugs. Accordingly, it may be more reasonable to switch to easy-to-use and well-tolerated maintenance therapy drugs (25). Capecitabine and S-1 are oral fluoropyrimidine agents, widely used in the treatment of solid tumors, and are potential maintenance therapy drugs to prevent disease progression. A significant improvement of 5-year DFS was obtained in women with early-stage triple-negative breast cancer after 1 year of low-dose capecitabine maintenance therapy following standard adjuvant therapy (17). In addition, another phase III RCT compared capecitabine maintenance therapy with observation in patients with advanced colorectal cancer treated with XELOX or FOLFOX (26). The results showed that the PFS was significantly longer in the capecitabine maintenance therapy group than in the observation group (6.43 months vs. 3.43 months, HR 0.54, *p* < 0.001), and the safety profiles of the two groups were comparable (26). Therefore, capecitabine may be an option for maintenance therapy after first-line treatment in patients with advanced malignancies. In our case, the patient received maintenance therapy with capecitabine for 8 months and subsequently switched to S-1 due to elevated CA199, obtaining a total of 11 months of DFS and no grade 3 or higher AEs. Our results suggest that maintenance therapy may result in longer disease control in high risk of relapse GBC individuals, and further studies are needed to confirm the role of maintenance therapy in advanced GBCs.

Although elevated serum CEA levels (>4.0 ng/ml) or CA199 levels (>20.0 U/ml) may be indicators of GBC, there is still a lack of tumor markers for predicting treatment response and disease recurrence in GBC. In the present case, the patient had an increase in CA199 before the lesion was detected on PET-CT and the CEA was increased. Moreover, CA199 was more closely associated with the remission of the disease than CEA and the imaging information during the whole treatment process. Therefore, we considered that monitoring of CA199 should be strengthened not only during but also after treatment, which may help us to detect disease recurrence in time and achieve better survival outcomes with early and active intervention.

Tumor-infiltrating lymphocytes (TILs) are heterogeneous lymphocytes that present in the tumor microenvironment, and mainly include CD8^+^ cytotoxic T lymphocytes, CD4^+^ T helper lymphocytes, and FoxP3^+^ Tregs (27). Several studies have demonstrated that CD4^+^ and CD8^+^ T-cell infiltration was associated with a better prognosis, whereas low numbers of CD8^+^ T cells were associated with poor outcomes in GBC patients (*p* = 0.02) (28, 29). Tregs are characterized by the secretion of TGF-β and IL-10, which contribute to an immunosuppressive environment and help tumor cells achieve immune escape. The presence of large amounts of Tregs in the GBC was significantly associated with poor OS (*p* = 0.04) (29). In addition, the binding of PD-1 and PD-L1 in the tumor microenvironment can inhibit T-cell activity and promote tumor cell evasion of immune surveillances (30). Thus, low PD-(L) 1 expression is associated with better prognosis (31, 32). Because GBC was a highly aggressive malignant tumor prone to local infiltration and hematogenous metastasis at an early stage, we performed a tumor microenvironment assay on the postoperative specimen of this patient, which might reflect the tumor immune microenvironment of the patient after recurrence to some extent. In this case, the patient had more CD4^+^ and CD8^+^ T-cell infiltration and fewer Foxp3^+^ T cells and PD-(L) 1^+^ cells, which may be the key to the patient’s long-term survival.

## Conclusion

We described an advanced GBC patient who achieved a CR after first-line treatment with nab-paclitaxel in combination with gemcitabine-cisplatin. He subsequently received maintenance therapy with fluorouracil analogs and achieved a DFS of 11 months. In addition, he remained responsive to nab-paclitaxel plus gemcitabine-cisplatin after the disease progressed. This impressive response highlighted the effectiveness of nab-paclitaxel for the treatment of GBC. We also reported for the first time the role of capecitabine or S-1 as maintenance therapy in GBC. Furthermore, our results demonstrated that CA199 was more sensitive than CEA or even PET-CT in predicting treatment response and recurrence. Our further IHC analysis revealed that the higher number of CD4^+^ and CD8^+^ T cells and the lower number of Tregs and PD-(L)1^+^ cells might be key reasons for the patient’s long-term survival. We expect that our report can act as a reference supporting the systematic treatment, monitoring, and prognosis determination of GBC patients.

## Data Availability Statement

The original contributions presented in the study are included in the article/supplementary material. Further inquiries can be directed to the corresponding author.

## Ethics Statement

Written informed consent was obtained from the individual for the publication of this case report and any potentially identifiable images or data included in this article.

## Author Contributions

TL conceived the study, performed the literature research, wrote the paper, and assessed figure and tables. QL collected the pathological samples and clinical data and confirmed the histological diagnosis. WZ performed the literature research and critically reviewed the paper. QZ supervised the project. All authors contributed to the article and approved the submitted version.

## Funding

This research was funded by 1.3.5 Project for Disciplines of Excellence, West China Hospital (ZYJC21042), Sichuan University for QZ.

## Conflict of Interest

The authors declare that the research was conducted in the absence of any commercial or financial relationships that could be construed as a potential conflict of interest.

## Publisher’s Note

All claims expressed in this article are solely those of the authors and do not necessarily represent those of their affiliated organizations, or those of the publisher, the editors and the reviewers. Any product that may be evaluated in this article, or claim that may be made by its manufacturer, is not guaranteed or endorsed by the publisher.

## References

[1] Valle JW Lamarca A Goyal L Barriuso J Zhu AX. New Horizons for Precision Medicine in Biliary Tract Cancers. Cancer Discov (2017) 7(9):943–62. doi: 10.1158/2159-8290.CD-17-0245 PMC5586506 28818953

[2] Nepal C Zhu B O'Rourke CJ Bhatt DK Lee D Song L. Integrative Molecular Characterisation of Gallbladder Cancer Reveals Micro-Environment-Associated Subtypes. J Hepatol (2021) 74(5):1132–44. doi: 10.1016/j.jhep.2020.11.033 PMC8058239 33276026

[3] Valle JW Kelley RK Nervi B Oh DY Zhu AX. Biliary Tract Cancer. Lancet (2021) 397(10272):428–44. doi: 10.1016/S0140-6736(21)00153-7 33516341

[4] Bray F Ferlay J Soerjomataram I Siegel RL Torre LA Jemal A. Global Cancer Statistics 2018: GLOBOCAN Estimates of Incidence and Mortality Worldwide for 36 Cancers in 185 Countries. CA Cancer J Clin (2018) 68(6):394–424. doi: 10.3322/caac.21492 30207593

[5] Song X Hu Y Li Y Shao R Liu F Liu Y. Overview of Current Targeted Therapy in Gallbladder Cancer. Signal Transduct Target Ther (2020) 5(1):230. doi: 10.1038/s41392-020-00324-2 33028805 PMC7542154

[6] Costi R Violi V Roncoroni L Sarli L. Gallbladder Cancer and Radical Surgery. Ann Surg (2008) 248(3):494; author reply 495–6. doi: 10.1097/SLA.0b013e318186061a 18791371

[7] Bridgewater J Galle PR Khan SA Llovet JM Park JW Patel T. Guidelines for the Diagnosis and Management of Intrahepatic Cholangiocarcinoma. J Hepatol (2014) 60(6):1268–89. doi: 10.1016/j.jhep.2014.01.021 24681130

[8] Baiu I Visser B. Gallbladder Cancer. Jama (2018) 320(12):1294. doi: 10.1001/jama.2018.11815 30264121

[9] Valle J Wasan H Palmer DH Cunningham D Anthoney A Maraveyas A. Cisplatin Plus Gemcitabine Versus Gemcitabine for Biliary Tract Cancer. N Engl J Med (2010) 362(14):1273–81. doi: 10.1056/NEJMoa0908721 20375404

[10] Frese KK Neesse A Cook N Bapiro TE Lolkema MP Jodrell DI. Nab-Paclitaxel Potentiates Gemcitabine Activity by Reducing Cytidine Deaminase Levels in a Mouse Model of Pancreatic Cancer. Cancer Discov (2012) 2(3):260–9. doi: 10.1158/2159-8290.CD-11-0242 PMC4866937 22585996

[11] Von Hoff DD Ervin T Arena FP Chiorean EG Infante J Moore M. Increased Survival in Pancreatic Cancer With Nab-Paclitaxel Plus Gemcitabine. N Engl J Med (2013) 369(18):1691–703. doi: 10.1056/NEJMoa1304369 PMC4631139 24131140

[12] Mizrahi JD Surana R Valle JW Shroff RT. Pancreatic Cancer. Lancet (2020) 395(10242):2008–20. doi: 10.1016/S0140-6736(20)30974-0 32593337

[13] Shroff RT Javle MM Xiao L Kaseb AO Varadhachary GR Wolff RA. Gemcitabine, Cisplatin, and Nab-Paclitaxel for the Treatment of Advanced Biliary Tract Cancers: A Phase 2 Clinical Trial. JAMA Oncol (2019) 5(6):824–30. doi: 10.1001/jamaoncol.2019.0270 PMC6567834 30998813

[14] Gay F Jackson G Rosiñol L Holstein SA Moreau P Spada S. Maintenance Treatment and Survival in Patients With Myeloma: A Systematic Review and Network Meta-Analysis. JAMA Oncol (2018) 4(10):1389–97. doi: 10.1001/jamaoncol.2018.2961 PMC6233774 30098165

[15] Gomez DR Blumenschein GR Jr Lee JJ Hernandez M Ye R Camidge DR. Local Consolidative Therapy Versus Maintenance Therapy or Observation for Patients With Oligometastatic Non-Small-Cell Lung Cancer Without Progression After First-Line Systemic Therapy: A Multicentre, Randomised, Controlled, Phase 2 Study. Lancet Oncol (2016) 17(12):1672–82. doi: 10.1016/S1470-2045(16)30532-0 PMC5143183 27789196

[16] Pujade-Lauraine E Ledermann JA Selle F Gebski V Penson RT Oza AM. Olaparib Tablets as Maintenance Therapy in Patients With Platinum-Sensitive, Relapsed Ovarian Cancer and a BRCA1/2 Mutation (SOLO2/ENGOT-Ov21): A Double-Blind, Randomised, Placebo-Controlled, Phase 3 Trial. Lancet Oncol (2017) 18(9):1274–84. doi: 10.1016/S1470-2045(17)30469-2 28754483

[17] Wang X Wang SS Huang H Cai L Zhao L Peng RJ. Effect of Capecitabine Maintenance Therapy Using Lower Dosage and Higher Frequency vs Observation on Disease-Free Survival Among Patients With Early-Stage Triple-Negative Breast Cancer Who Had Received Standard Treatment: The SYSUCC-001 Randomized Clinical Trial. JAMA (2021) 325(1):50–8. doi: 10.1001/jama.2020.23370 PMC7729589 33300950

[18] Simkens LH van Tinteren H May A ten Tije AJ Creemers GJ Loosveld OJ. Maintenance Treatment With Capecitabine and Bevacizumab in Metastatic Colorectal Cancer (CAIRO3): A Phase 3 Randomised Controlled Trial of the Dutch Colorectal Cancer Group. Lancet (2015) 385(9980):1843–52. doi: 10.1016/S0140-6736(14)62004-3 25862517

[19] Abdel-Rahman O Elsayed Z Elhalawani H. Gemcitabine-Based Chemotherapy for Advanced Biliary Tract Carcinomas. Cochrane Database Syst Rev (2018) 4(4):Cd011746. doi: 10.1002/14651858.CD011746.pub2 29624208 PMC6494548

[20] Kim ST Kang JH Lee J Lee HW Oh SY Jang JS. Capecitabine Plus Oxaliplatin Versus Gemcitabine Plus Oxaliplatin as First-Line Therapy for Advanced Biliary Tract Cancers: A Multicenter, Open-Label, Randomized, Phase III, Noninferiority Trial. Ann Oncol (2019) 30(5):788–95. doi: 10.1093/annonc/mdz058 30785198

[21] Morizane C Okusaka T Mizusawa J Katayama H Ueno M Ikeda M. Combination Gemcitabine Plus S-1 Versus Gemcitabine Plus Cisplatin for Advanced/Recurrent Biliary Tract Cancer: The FUGA-BT (JCOG1113) Randomized Phase III Clinical Trial. Ann Oncol (2019) 30(12):1950–8. doi: 10.1093/annonc/mdz402 31566666

[22] Lindner JL Loibl S Denkert C Ataseven B Fasching PA Pfitzner BM. Expression of Secreted Protein Acidic and Rich in Cysteine (SPARC) in Breast Cancer and Response to Neoadjuvant Chemotherapy. Ann Oncol (2015) 26(1):95–100. doi: 10.1093/annonc/mdu487 25355716

[23] Cheng CT Chu YY Yeh CN Huang SC Chen MH Wang SY. Peritumoral SPARC Expression and Patient Outcome With Resectable Intrahepatic Cholangiocarcinoma. Onco Targets Ther (2015) 8:1899–907. doi: 10.2147/ott.s78728 PMC4524580 26251613

[24] Von Hoff DD Ramanathan RK Borad MJ Laheru DA Smith LS Wood TE. Gemcitabine Plus Nab-Paclitaxel Is an Active Regimen in Patients With Advanced Pancreatic Cancer: A Phase I/II Trial. J Clin Oncol (2011) 29(34):4548–54. doi: 10.1200/JCO.2011.36.5742 PMC3565012 21969517

[25] Gligorov J Doval D Bines J Alba E Cortes P Pierga JY. Maintenance Capecitabine and Bevacizumab Versus Bevacizumab Alone After Initial First-Line Bevacizumab and Docetaxel for Patients With HER2-Negative Metastatic Breast Cancer (IMELDA): A Randomised, Open-Label, Phase 3 Trial. Lancet Oncol (2014) 15(12):1351–60. doi: 10.1016/S1470-2045(14)70444-9 25273343

[26] Luo HY Li YH Wang W Wang ZQ Yuan X Ma D. Single-Agent Capecitabine as Maintenance Therapy After Induction of XELOX (or FOLFOX) in First-Line Treatment of Metastatic Colorectal Cancer: Randomized Clinical Trial of Efficacy and Safety. Ann Oncol (2016) 27(6):1074–81. doi: 10.1093/annonc/mdw101 26940686

[27] Rodrigues PM Olaizola P Paiva NA Olaizola I Agirre-Lizaso A Landa A. Pathogenesis of Cholangiocarcinoma. Annu Rev Pathol (2021) 16:433–63. doi: 10.1146/annurev-pathol-030220-020455 33264573

[28] Goeppert B Frauenschuh L Zucknick M Stenzinger A Andrulis M Klauschen F. Prognostic Impact of Tumour-Infiltrating Immune Cells on Biliary Tract Cancer. Br J Cancer (2013) 109(10):2665–74. doi: 10.1038/bjc.2013.610 PMC3833207 24136146

[29] Kitano Y Okabe H Yamashita YI Nakagawa S Saito Y Umezaki N. Tumour-Infiltrating Inflammatory and Immune Cells in Patients With Extrahepatic Cholangiocarcinoma. Br J Cancer (2018) 118(2):171–80. doi: 10.1038/bjc.2017.401 PMC5785749 29123259

[30] Kumagai S Togashi Y Kamada T Sugiyama E Nishinakamura H Takeuchi Y. The PD-1 Expression Balance Between Effector and Regulatory T Cells Predicts the Clinical Efficacy of PD-1 Blockade Therapies. Nat Immunol (2020) 21(11):1346–58. doi: 10.1038/s41590-020-0769-3 32868929

[31] Chida K Kawazoe A Kawazu M Suzuki T Nakamura Y Nakatsura T. A Low Tumor Mutational Burden and PTEN Mutations Are Predictors of a Negative Response to PD-1 Blockade in MSI-H/dMMR Gastrointestinal Tumors. Clin Cancer Res (2021). doi: 10.1158/1078-0432.CCR-21-0401 33926917

[32] Lu J-C Zeng H-Y Sun Q-M Meng Q-N Huang X-Y Zhang P-F. Distinct PD-L1/PD1 Profiles and Clinical Implications in Intrahepatic Cholangiocarcinoma Patients With Different Risk Factors. Theranostics (2019) 9(16):4678–87. doi: 10.7150/thno.36276 PMC6643449 31367249

